# Effects of Variability in Glycemic Indices on Longevity in Chinese Centenarians

**DOI:** 10.3389/fnut.2022.955101

**Published:** 2022-07-08

**Authors:** Sheng-han Ji, Chen Dong, Rou Chen, Chen-chen Shen, Jing Xiao, Yun-juan Gu, Jian-lin Gao

**Affiliations:** ^1^Department of Endocrinology and Metabolism, Affiliated Hospital of Nantong University, Nantong, China; ^2^Medical School of Nantong University, Nantong University, Nantong, China; ^3^Research Center of Gerontology and Longevity, Affiliated Hospital of Nantong University, Nantong, China; ^4^Department of Cardiology, Rugao Bo'ai Branch of Nantong University Affiliated Hospital, Nantong, China; ^5^Department of Epidemiology and Medical Statistics, School of Public Health, Nantong University, Nantong, China; ^6^Department of Health Medicine, Affiliated Hospital of Nantong University, Nantong, China

**Keywords:** centenarians, continuous glucose monitoring, glucose variability, hypoglycemia, longevity

## Abstract

**Background:**

Large fluctuations in blood glucose levels greatly impact the health and life span of elderly individuals. This study describes the characteristics of variability in glycemic indices in centenarians with the aim of emphasizing the importance of glycemic variability in elderly people.

**Methods:**

We recruited individuals from Rugao City, Jiangsu Province, China from April 2020 to May 2021. The study cohort included 60 centenarians and 60 first-generation offspring, as well as 20 randomly selected non-cohabitant control individuals aged 60–80 years. A FreeStyle Libre H (hospital version) continuous glucose monitoring (CGM) device (Abbott Ireland UK) was used to measure glycemic variability. The indices measured included the time in target glucose range (TIR), time below target glucose range (TBR), time above target glucose range (TAR), mean amplitude of glycemic excursions (MAGE), mean of daily differences (MODD), coefficient of variation (CV), standard deviation of blood glucose (SDBG), continuous overlapping net glycemic action (CONGA), glucose management indicator (GMI) and estimated glycated hemoglobin (eHbA1c). Logistic regression was used to analyze the association between glycemic variability and longevity.

**Results:**

Mean blood glucose (MBG), eHbA1c, GMI, mean fasting plasma glucose (M-FPG) and CONGA were lower in the centenarian group (*p* all < 0.05). PPGE-2 was higher in the control group than that measured in the centenarian and first-generation offspring groups (*p* < 0.05). There were no differences between the groups in MAGE, MODD, MAG, or TIR (*p* > 0.05). The risk of not achieving longevity increased with each one unit increase in MBG by 126% [2.26 (1.05–4.91)], eHbA1c by 67% [1.67 (1.03–2.72)], GMI by 568% [6.68 (1.11–40.30)], M-FPG by 365% [4.65 (1.57–13.75)], M-PPG1h by 98% [1.98 (1.18–3.31)], CONGA1 by 102% [2.02 (1.01–4.06)], Li by 200% [3.00 (1.04–8.61)], and PPGE-2 by 150% [2.50 (1.39–4.50)]. However, the risk of achieving longevity decreased with each unit increase of LBGI by 53% [0.47 (0.28–0.80)], ADRR by 60% [0.40 (0.18–0.86)], and TBR by 11% [0.89 (0.80–0.98)].

**Conclusion:**

Fluctuation in blood glucose levels in centenarians is relatively small. Maintaining an average blood glucose level and keeping blood glucose fluctuations in the normal range is conducive to longevity.

## Introduction

Diabetes mellitus (DM) is a chronic metabolic disease characterized by a long course of disease and health complications, which can shorten the lifespan of middle-aged and elderly individuals (1). The World Health Organization (WHO) has reported that DM is one of the first ten major causes of death and disability globally (2). In China, ~1.4 million people die of DM every year, representing one death every 22 seconds (2). DM is therefore an important determinant of reduced longevity (2). Analyzing the blood glucose status of centenarians can help identify the associations between blood glucose and longevity, thereby providing suggestions for clinical blood glucose control that will facilitate healthy aging. A study in Italian centenarians showed that fasting plasma glucose (FPG) levels were significantly lower in centenarians than in cohabitants (3). The results of the China Hainan centenary cohort study and the China Hainan senior cohort study also showed that blood glucose levels and the prevalence of diabetes were lower in centenarians than those in non-centenarians (4), while another study in Polish centenarians reported that their blood glucose levels were significantly lower than in elderly individuals (5). A previous study on glycemic control only focused on periodic glucose measures and average level of glycated hemoglobin (HbA1c). However, the relative importance of glycemic variation over time has attracted increasing attention, although only a few relevant studies have been carried out in centenarians. There is evidence that compared with hyperglycemia, an increase in the variability of glucose levels may damage vascular endothelial cells and that such changes are associated with a higher risk of cardio-cerebrovascular and microvascular complications (6, 7). Continuous glucose monitoring (CGM) may therefore be more applicable to accurately evaluating variability in glucose levels. Research has shown that HbA1c levels are reduced to a greater extent after CGM compared with that achieved by glucometer testing (8). It has also been shown that CGM can markedly reduce the incidence of hypoglycemia in elderly people (9). However, the use of CGM in centenarians has not been reported extensively in the current literature. To address this situation, we recruited centenarians from Rugao City, an area famous for the longevity of its population (10). The first-generation of the centenarians' offspring and non-longevity individuals were recruited as control groups. A comprehensive examination of the relationship between glycemic variation and longevity was performed in order to provide a clinical basis to facilitate healthy aging in the population.

## Materials and Methods

### Study Participants

We recruited individuals from Rugao City, Jiangsu Province China from April 2020 to May 2021. The cohort included 60 centenarians, 60 first-generation offspring, and 20 non-cohabitant controls aged 60–80 years from the same region who were selected randomly during the same period. Each individual was asked to complete a household survey. Exclusion criteria included acute diabetic complications, being long-term bedridden, secondary DM, malignancies, cognitive impairment, severe infection, recent trauma, surgery or other emergencies, severe systemic diseases, individuals currently using glucocorticoids, and those who did not have sufficient time to carry out continuous glucose monitoring (CGM). The final cohort included 53 centenarians, 53 first-generation offspring cohabitants, and 20 controls. The demographic characteristics of these three groups are summarized in Table 1. The study followed the Declaration of Helsinki thoroughly and was approved by the medical research ethics committee of the Second Affiliated Hospital of Nantong University.

**Table 1:** Clinical characteristics of the participants, according to group assignment.

**Parameter**	**Centenarian group**	**First-generation offspring group**	**Control group**	***P***
	**(*n* = 53)**	**(*n* = 53)**	**(*n* = 20)**	
Age (yr)	100.0 (100.0, 101.0)	69.0 (63.0, 75.0)^a1^	69.0 (65.0, 77.0)^b1^	<0.001
SBP (mmHg)	155.0 (134.3, 178.8)	142.0 (124.5, 156.0)^a2^	130.3 (121.0, 152.4)^b2^	0.001
DBP (mmHg)	73.5 (65.3, 84.0)	78.5 (71.8, 87.8)	73.0 (66.6, 85.9)	0.053
Gender				
Male	12 (22.6)	39 (73.6)^a1^	7 (35.0)^c2^	<0.001
Female	41 (77.4)	14 (26.4)	13 (65.0)	<0.001
Marital status *n* (%)				
Married	1 (1.9)	47 (88.7)^a1^	19 (95.0)^b1^	<0.001
Single	52 (98.1)	6 (11.3)	1 (5.0)	<0.001
Educational level *n* (%)				
Illiterate	40 (75.5)	3 (5.7)^a1^	8 (40.0)^b2^^c1^	<0.001
Primary	11 (20.7)	21 (39.6)	8 (40.0)	<0.001
Junior	1 (1.9)	21 (39.6)	3 (15.0)	
Senior and above	1 (1.9)	8 (15.1)	1 (5.0)	
Hypertension *n* (%)				
Yes	17 (32.7)	20 (37.7)	6 (30.0)	0.794
No	35 (67.3)	33 (62.3)	14 (70.0)	0.794
DM *n* (%)				
Yes	2 (3.8)	5 (9.4)	1 (5.0)	0.63
No	51 (96.2)	48 (90.6)	19 (95.0)	0.63
Smoking *n* (%)				
Never	43 (81.1)	26 (50.0)^a1^	10 (50.0)^b2^	
Quit	7 (13.2)	12 (23.1)	4 (20.0)	0.003
Yes	3 (5.7)	14 (26.9)	6 (30.0)	
Drinking *n* (%)				
Never	37 (71.1)	21 (40.4)^a1^	8 (40.0)^b2^	
Quit	8 (15.4)	5 (9.6)	4 (20.0)	0.001
Yes	7 (13.5)	26 (50.0)	8 (40.0)	

a1 *: centenarian group vs. first-generation offspring group, p < 0.001*;

a2 *: centenarian group vs. first-generation offspring group, p < 0.05*;

b1 *: centenarian group vs. control group, p < 0.001*;

b2 *: centenarian group vs. control group, p < 0.05*;

c1 *: first-generation offspring group vs. control group, p < 0.001*;

c2 *: first-generation offspring group vs. control group, p < 0.05*.

### Data Acquisition

#### Basic Data Collection

Demographic data including gender, age, educational level, marital and fertility history, smoking and drinking status were collected using professional medical questionnaires.

#### Glucose Monitoring

Continuous blood glucose was measured by a flash glucose monitoring (FGM) and CGM system (Abbott, FreeStyle Libre H, hospital version) (11) using standard protocols. The measurements were recorded over at least 12 consecutive 24-h periods, with the first and last 24-h periods excluded from the analysis.

#### CGM Index

Mean blood glucose (MBG), standard deviation of blood glucose (SDBG), coefficient of variation (CV), estimated glycated hemoglobin (eHbA1c), glucose management indicator (GMI), mean-fasting plasma glucose (M-FPG), mean-postprandial plasma glucose (M-PPG), M-PPG-1h, M-PPG-2h, M-PPG-3h, J index, M value, low blood glucose index (LBGI), high blood glucose index (HBGI), average daily risk range (ADRR), continuous overlapping net glycemic action (CONGA), mean absolute glucose (MAG), lability index (Li), postprandial plasma glycemic excursion (PPGE), PPGE-1, PPGE-2, PPGE-3, mean amplitude of glycemic excursion (MAGE), largest amplitude of glycemic excursion (LAGE), mean of daily differences (MODD), time in the target glucose range (TIR), time below the target glucose range (TBR), and time above the target glucose range (TAR). The definition and interpretation of these indices are shown in Supplementary Table 1.

#### Diagnostic Criteria

The diagnostic criteria for DM (12, 13) used in the study were as follows: (1) polydipsia, polyuria, polyphagia, loss of body weight, and a random blood glucose level ≥11.1 mmol/L, (2) fasting plasma glucose (FPG) ≥7.0 mmol/L, (3) oral glucose tolerance test (OGTT) 2-h postprandial blood glucose ≥11.1 mmol/L. Hypertension: elevation of systemic arterial pressure in the resting state and a doctor's office blood pressure ≥140/90 mmHg (14).

#### Statistical Analysis

Normally distributed continuous numerical variables were expressed as mean ± standard deviation (SD). For each group, the differences were analyzed using *t*-tests. Non-normally distributed data were first logarithmically transformed and if still skewed, the Mann-Whitney U or Kruskal-Wallis tests were used to test the differences, expressed as median (interquartile range). Categorical variables were expressed as *n* (%) and analyzed using Chi-square tests. The association between longevity and blood glucose indices were analyzed by logistic regression analyses. Because the genetic relationship between centenarians and their offspring would affect the results of the correlation analysis in the study, the logistic regression analyses were only conducted between centenarian and control groups, who did not have a genetic relationship. The *P*-values were two-tailed, with *P* < 0.05 considered statistically significant. IBM SPSS for Windows, version 26.0 software was used for the statistical analyses.

## Results

### Study Cohort

Age and systolic blood pressure were significantly higher in the centenarian group than in the first-generation offspring and control groups (*P* < 0.05). The centenarian and control group had lower proportions of males and married individuals than the first-generation offspring group (*P* < 0.001), while the illiteracy rate was the highest in the centenarian group (*P* < 0.001). The centenarian group was also more likely to include non-smokers or drinkers (*P* < 0.05). There were no differences in diastolic blood pressure, income, hypertension, and DM status between the three groups (*P* > 0.05) (Table 1).

### Comparison of Continuous Blood Glucose Indexes Among the Three Groups

In the centenarian group, MBG, CONGA1, eHbA1c, GMI, M-FPG, and M-PPG1h were lower (*P* < 0.05) and LBGI and ADRR higher (*P* < 0.001) than those measured in the first-generation offspring and control groups. The control group had higher PPGE-2 than the centenarian and first-generation offspring groups (*P* = 0.002). There was no significant difference in TIR (*P* > 0.05) between the three groups (Table 2).

**Table 2:** Continuous blood glucose parameters according to group assignment.

**Parameter**	**Centenarian group**	**First-generation offspring group**	**Control group**	***P***
	**(*n* = 53)**	**(*n* = 53)**	**(*n* = 20)**	
MBG (mmol/L)	5.31 (4.8, 5.6)	5.6 (5.3, 5.9)^a1^	5.7 (5.1, 6.2)^b1^	0.001
SDBG (mmol/L)	1.4 ± 0.4	1.4 ± 0.5	1.6 ± 0.6	0.398
CV	0.2 ± 0.06	0.2 ± 0.06^a2^	0.3 ± 0.06	0.023
eHbA1c (%)	5.9 (5.1, 6.3)	6.4 (5.8, 6.9) ^a1^	6.5 (5.5, 7.2) ^b2^	0.001
GMI (mmol/mol)	37.6 (35.4, 38.9)	39.3 (37.6, 40.8) ^a1^	39.7 (36.8, 41.9) ^b2^	0.001
M-FPG (mmol/L)	4.1 (3.9, 4.6)	4.9 (4.5, 5.3)^a1^	4.8 (4.4, 5.1)^b1^	<0.001
M-PPG1h (mmol/L)	4.4 (4.0, 5.3)	5.2 (4.7, 5.8) ^a1^	4.9 (4.6, 6.9)^b2^	<0.001
M-PPG2h (mmol/L)	5.7 ± 1.6	6.4 ± 1.8	6.6 ± 1.9	0.064
M-PPG3h (mmol/L)	6.2 ± 1.7	6.8 ± 2.2	6.4 ± 1.7	0.238
J index (mmol/L)	15.1 ± 5.2	18.5 ± 12.0	20.3 ± 12.7	0.080
M Value	29.8 (16.8, 46.2)	21.5 (15.5, 39.1)	33.2 (16.1, 64.2)	0.318
LBGI	3.7 ± 2.1	2.0 ± 1.7^a1^	2.1 ± 1.3 ^b2^	<0.001
HBGI	0.2 (0.1, 0.6)	0.1 (0.1, 0.6)	0.6 (0.1, 1.2)	0.246
ADRR	33.2 (32.9, 33.8)	32.7 (32.4, 33.2)^a1^	32.7 (32.2, 33.4)^b2^	<0.001
CONGA1 (mmol/L)	5.5 (5.0, 5.8)	5.8 (5.4, 6.1)^a2^	5.9 (5.2, 6.5)^b2^	0.006
MAG (mmol/L)	2.94 ± 0.92	3.00 ± 1.16	3.21 ± 1.07	0.256
Li	1.3 (1.1, 1.6)	1.5 (1.0, 2.0)	1.6 (1.2, 2.)	0.158
PPGE (mmol/L)	2.7 ± 0.9	2.7 ± 0.9	2.9 ± 1.1	0.656
PPGE-1 (mmol/L)	3.3 ± 1.5	3.2 ± 1.7	2.8 ± 1.5	0.400
PPGE-2 (mmol/L)	2.1 ± 1.1	2.3 ± 1.3	3.2 ± 1.3^b2^^c1^	0.002
PPGE-3 (mmol/L)	2.7 ± 1.2	2.6 ± 1.1	2.8 ± 1.2	0.811
MAGE (mmol/L)	1.7 ± 0.4	1.7 ± 0.5	1.8 ± 0.5	0.707
LAGE (mmol/L)	5.3 ± 1.4	5.3 ± 1.9	5.7 ± 1.8	0.615
MODD (mmol/L)	0.9 ± 0.3	0.9 ± 0.4	1.1 ± 0.4	0.057
TIR	0.9 (0.8, 0.9)	0.9 (0.8, 0.9)	0.8 (0.8, 0.9)	0.094
TBR	0.06 ± 0.11	0.07 ± 0.09	0.10 ± 0.13	0.379
TAR	0.03 ± 0.09	0.02 ± 0.06	0.07 ± 0.20	0.676

a1 *: centenarian group vs. first-generation offspring group, p < 0.001*;

a2 *: centenarian group vs. first-generation offspring group, p < 0.05*;

b1 *: centenarian group vs. control group, p < 0.001*;

b2 *: centenarian group vs. control group, p < 0.05*;

c1 *: first-generation offspring group vs. control group, p < 0.05*.

### Association Between Blood Glucose Fluctuation and Anti-longevity

The risk of not achieving longevity increased with each unit increase in the blood glucose indices MBG by 126% [OR (95%CI): 2.26 (1.05–4.91)], eHbA1c by 67% [OR (95%CI): 1.67 (1.03–2.72)], GMI by 568% [OR (95%CI): 6.68 (1.11–40.30)], M-FPG by 365% [OR (95%CI): 4.65 (1.57–13.75)], and M-PPG1h by 98% [OR (95%CI): 1.98 (1.18–3.31)]. The risk of not achieving longevity also increased with each unit increase in CONGA1 by 102% [OR (95%CI): 2.02 (1.01–4.06)], Li by 200% [OR (95%CI): 3.00 (1.04–8.61)], and PPGE-2 by 150% [OR (95%CI): 2.50 (1.39–4.50)]. However, the risk of not achieving longevity decreased with each unit increase of LBGI by 53% [OR (95%CI): 0.47 (0.28–0.80)], ADRR by 60% [OR (95%CI): 0.40 (0.18–0.86)], and TBR by 11% [OR (95%CI): 0.89 (0.80–0.98)]. Among these indicators of blood glucose variability, GMI had the largest OR value and therefore showed the strongest correlation with longevity (Table 3). However, associations between not achieving longevity and SDBG, M-PPG2h, M-PPG3h, J index, M Value, HBGI, MAG, Li, PPGE, PPGE-1, PPGE-3, MAGE, LAGE, MODD, TIR, TBR, TAR were not found in this study.

**Table 3:** Logistic regression analysis of not achieving longevity and CGM indices.

**Parameter**	**OR (95 % CI)^**a**^**	***P***	**OR (95 % CI)^**b**^**	***P***
MBG (mmol/L)	1.93 (1.11–3.36)	0.020	2.26 (1.05–4.91)	0.038
SDBG (mmol/L)	1.86 (0.66–5.26)	0.241	1.27 (0.34–4.69)	0.724
CV (%)	0.97 (0.89–1.06)	0.460	0.90 (0.80–1.01)	0.086
eHbA1c (%)	1.51 (1.07–2.14)	0.020	1.67 (1.03–2.72)	0.038
GMI (mmol/mol)	4.61 (1.28–16.69)	0.020	6.68 (1.11–40.30)	0.038
M-FPG (mmol/L)	3.33 (1.46–7.62)	0.004	4.65 (1.57–13.75)	0.005
M-PPG1h (mmol/L)	1.75 (1.15–2.67)	0.010	1.98 (1.18–3.31)	0.010
M-PPG2h (mmol/L)	1.32 (0.99–1.76)	0.060	1.22 (0.88–1.69)	0.238
M-PPG3h (mmol/L)	1.06 (0.78–1.44)	0.710	0.98(0.65–1.48)	0.936
J index (mmol/L)	1.08 (1.01–1.16)	0.038	1.09 (0.98–1.20)	0.100
M Value	1.01 (1.00–1.02)	0.098	1.01 (1.00–1.02)	0.262
LBGI	0.58 (0.40–0.85)	0.005	0.47 (0.28–0.80)	0.005
HBGI	1.51 (0.98–2.35)	0.064	1.55 (0.84–2.88)	0.161
ADRR	0.49 (0.28–0.85)	0.012	0.40 (0.18–0.86)	0.020
CONGA1 (mmol/L)	1.81 (1.09–3.01)	0.023	2.02 (1.01–4.06)	0.050
MAG (mmol/L)	1.33 (0.78–2.26)	0.292	1.22 (0.63–2.35)	0.553
Li	2.69 (1.12–6.45)	0.027	3.00 (1.04–8.61)	0.041
PPGE (mmol/L)	1.27 (0.74–2.19)	0.387	1.19 (0.61–2.34)	0.603
PPGE-1 (mmol/L)	0.76 (0.52–1.12)	0.164	0.54 (0.31–0.97)	0.040
PPGE-2 (mmol/L)	2.14 (1.34–3.42)	0.002	2.50 (1.39–4.50)	0.002
PPGE-3 (mmol/L)	1.08 (0.68–1.74)	0.736	1.03 (0.60–1.78)	0.910
MAGE (mmol/L)	1.41 (0.42–4.68)	0.580	1.05 (0.25–4.45)	0.943
LAGE (mmol/L)	1.22 (0.85–1.73)	0.279	1.12 (0.73–1.72)	0.612
MODD (mmol/L)	8.03 (1.52–42.44)	0.014	6.38 (0.82–49.60)	0.077
TIR (%)	1.02 (0.98–1.07)	0.287	1.05 (0.99–1.11)	0.103
TBR (%)	0.91 (0.84–0.99)	0.020	0.89 (0.80–0.98)	0.018
TAR (%)	1.09 (0.99–1.19)	0.054	1.10 (0.97–1.24)	0.130

a *: Unadjusted;*

b *: Adjusted for systolic blood pressure, gender, marital status and educational level, smoking and drinking status*.

## Discussion

The results of this study showed that the CGM indices (MBG, eHbA1c, GMI, M-FPG, M-PPG1h, and CONGA1) were significantly lower in centenarians than in first-generation offspring and control groups. The centenarian group also had significantly higher LBGI and ADRR than the first-generation offspring and control groups. The risk of not achieving longevity increased with each one unit increase of MBG (126%), eHbA1c (67%), GMI (568%), M-FPG (365%), M-PPG1h (98%), CONGA1 (102%), Li (200%), and PPGE-2 (150%), although decreased with each one unit increase of LBGI (53%), ADRR (60%), and TBR (11%).

By providing information on glucose trends and measuring fluctuations, CGM provides a better tool for glucose management, treatment guidance, and patient motivation (15). As shown in previous studies, marked blood glucose fluctuations cause greater harm than persistent hyperglycemia (16). Such fluctuations by generating reactive oxygen species (ROS), vascular endothelial cell damage, and inflammation (17–19) can result in kidney damage, diabetic retinopathy, vascular damage, and pancreatic β-cell dysfunction (20–24), thereby limiting individual lifespan. Mean blood glucose levels and fluctuations are associated significantly with all-cause mortality, with high levels resulting in reduced life expectancy (25, 26). However, recent studies on longevity have focused mainly on the effect of point blood glucose measurements, and have shown that FPG levels and DM-associated morbidity are lower in centenarians than in non-centenarians (4). It has also been reported that there is an association between HbA1c and risk of all-cause mortality (27). In recent years, many CGM indices have been used to describe blood glucose variability with evidence showing that MBG and SDBG reflect blood glucose variability, while MBG affects HbA1c level (28). TIR, MOOD, CV, CONGA, and Li have also been shown to reflect daily blood glucose variability (29–31). LBGI and ADRR and TBR are better markers of hypoglycemia and are known to be associated with a greater risk of developing this condition (5, 32). However, there are fewer comprehensive indices for CGM and many gaps in research remain in the use of CGM in centenarians.

Using dynamic blood glucose monitoring, we found that: (1) blood glucose parameters including MGB, eHbA1c, GMI, and M-FPG were lowest in the centenarian group; (2) there was no difference in blood glucose fluctuation parameters such as MAGE, MODD, and MAG among the three groups; (3) CONGA1 was lowest in the centenarian group, PPGE-2 was higher in the control group than in the centenarian and sub-generation groups, and TIR which reflects blood glucose compliance time was the same in the three groups. We also found that MBG, eHbA1c, GMI, M-FPG, M-PPG1h, CONGA1, Li, and PPGE-2 were associated with not achieving longevity, indicating that increased blood glucose levels and fluctuations are not conducive to longevity.

LBGI and ADRR were highest in the centenarian group suggesting a higher risk of hypoglycemia than in the first-generation offspring and control groups. Increases in LBGI, ADRR, and TBR were associated with an increased likelihood of longevity. To further examine these associations, we divided the centenarian TBR into 2 groups according to their hypoglycemia status (<3.0 mmol/L and 3.1–3.9 mmol/L) and found that in the range of 3.1–3.9 mmol/L the rate of TBR was only 6.94%. TBR in the <3.0 mmol/L group was close to zero, indicating an absence of symptomatic hypoglycemia. Our results also showed that centenarians had the lowest blood glucose levels of the three groups, suggesting that these lower levels were conducive to longevity. The results are shown in Supplementary Table 2.

The cross-sectional nature of our study limits our ability to definitively identify the causal links between the measured variables. However, given the potential importance of these findings, we consider that follow-up cohort studies should be carried out to further test these relationships. In addition, as this study was conducted during the COVID-19 pandemic, the sample size of the control group was smaller than would be normally possible. We are therefore currently addressing this situation with additional recruitment which will allow for independent validation of the outcomes.

Finally, on the basis of these data, we strongly encourage clinicians to monitor blood glucose dynamics in addition to obtaining “point” blood glucose measurements by utilizing CGM. In addition, therapy should be oriented toward reducing the magnitude of glycemic excursions, which may help patients to achieve better health and result in them living longer.

Glucose metabolism disorders can lead to a series of complications, such as microvascular pathologies of the nervous, renal, and vision systems and an increased risk of adverse macrovascular cardiovascular outcomes,that then lead to an increase in mortality and a reduction in life expectancy (33). Rozing's study show that blood glucose homeostasis of centenarian offspring was better than that of a control group (non-centenarian offspring) (34). Therefore, maintaining the steady state of glucose metabolism is conducive to realization of healthy aging. The use of continuous blood glucose monitoring can effectively reduce the average blood glucose and occurrence of adverse events (such as hypoglycemia), thereby improving the stability of blood glucose levels (35).

The mechanism of mild blood glucose fluctuations in centenarians is unclear. Paolisso's study showed that the level of insulin resistance in centenarians was low, and the insulin resistance was related closely to the occurrence of type 2 diabetes and fluctuations in blood glucose levels (36). Although insulin sensitivity will decrease with an increase in age due to a decrease in muscle content and associated weakness (37) some studies have reported that insulin sensitivity in centenarians was higher (38, 39). This may also be a reason why the blood glucose status of centenarians is relatively stable. However, the current study was a household survey, and blood sampling involved household blood collection. Due to the limited site and laboratory conditions, an oral glucose tolerance test (OGTT) and euglycemic glucose clamp could not be conducted. However, TyG, as a new index to measure insulin resistance, also has certain credibility (40). The results in Supplementary Table 4 show that for each unit increase in TyG, the risk of not achieving longevity increased by 73% [OR (95% CI):1.73 (1.14,2.64)]. This result also indicates that high insulin resistance is not conducive to longevity.

## Data Availability Statement

The raw data supporting the conclusions of this article will be made available by the authors, without undue reservation.

## Ethics Statement

The studies involving human participants were reviewed and approved by the Medical Research Ethics Committee of Affiliated Hospital of Nantong University (2019-K045). The patients/participants provided their written informed consent to participate in this study.

## Author Contributions

J-lG and Y-jG contributed to the design of the study and revised the manuscript. S-hJ and CD conducted the study, collected the data, explain the data, and completed the proof. RC and C-cS collected the data. JX analyzed the data. All authors have read and approved the final manuscript.

## Funding

The study was supported by the Science and Technology Support Program of Nantong (MS22019005 and MS12020015).

## Conflict of Interest

The authors declare that the research was conducted in the absence of any commercial or financial relationships that could be construed as a potential conflict of interest.

## Publisher's Note

All claims expressed in this article are solely those of the authors and do not necessarily represent those of their affiliated organizations, or those of the publisher, the editors and the reviewers. Any product that may be evaluated in this article, or claim that may be made by its manufacturer, is not guaranteed or endorsed by the publisher.
