# Supplementary material for: Effects of Variability in Glycemic Indices on Longevity in Chinese Centenarians
Source: Front Nutr. 2022 Jul 8;9:955101. doi: 10.3389/fnut.2022.955101 (PMC9307500; doi:10.3389/fnut.2022.955101)
Supplement: Supplementary file 1 [file Table_1.pdf]

Supplementary Table 1. Description of the parameters of the continuous glucose monitoring device

Variability measured	Formula	Explanation of symbols	Clinical meaning
ADRR	$\text{ADRR} = \frac{1}{N}\sum_{t=1}^{N}[\text{LR+HR}]$	$x_i = 1.509 \times \{[\ln G_i]^{1.084} - 5.381\}^2$<br>N =total number of blood glucose measurements<br>LR = hypoglycemia risk value<br>HR = risk value of hyperglycemia<br>x_i = converted blood glucose value	Reacts to the risk of hyperglycemia and hypoglycemia.
MAG	$\text{MAG} = \frac{\sum_{n=1}^{N-1}\lvert G_n - G_{n+1}\rvert}{T}$	G = measurement of blood sugar level<br>N = total number of blood glucose measurements in two weeks<br>T = total measurement time (hours)	The fluctuation in total blood sugar value over unit time.
MODD	$\text{MODD} = \frac{\sum_{t=t_1}^{t_k}(G_t - G_{t-1440})}{k}$	k = number of matched blood sugars<br>Gt = blood glucose monitoring value<br>Gt-1440=blood glucose monitoring value 1 day before matching<br>Gt	Response to daytime blood sugar fluctuations.
SD	$\sqrt{\frac{\sum(x_i - \bar{x})^2}{k-1}}$	x_i = individual observation<br>$\bar{x}$ = mean of observations<br>k =number of observations	For assessing intraday glucose variability.
CV	$\frac{s}{\bar{x}}$	s = standard deviation<br>$\bar{x}$ = mean of observations	Assesses intraday variability of serum glucose.
MAGE	$\sum_{\text{if } \lambda > v}\frac{\lambda}{n}$	λ = each blood glucose increase or decrease<br>(nadir peak or peak nadir)<br>n = number of observations<br>v = 1SD of mean glucose for 24-h period	The variation around a mean glucose value.
LBGI	$\frac{1}{N}\sum_{i=1}^{N} rl(x_i)$	$f(\text{BG}) = 1.509 \times [(\ln(\text{BG}))^{1.084} - 5.381]$for BG in mg/dL<br>$f(\text{BG}) = 1.509 \times [(\ln(18\times\text{BG}))^{1.084} - 5.381]$for BG in $\frac{\text{mmol}}{\text{L}}$	Risk of hypoglycemia.

Supplementary Table 1 continued

Variability measured	Formula	Explanation of symbols	Clinical meaning
HBGI	$\frac{1}{N}\sum_{i=1}^{N} rh(x_i)$	$r(\mathrm{BG}) = 10 \times f(\mathrm{BG})^2$<br>$rl(\mathrm{BG}) = r(\mathrm{BG})$ if $f(\mathrm{BG}) < 0$ and 0 otherwise<br>$rh(\mathrm{BG}) = r(\mathrm{BG})$ if $f(\mathrm{BG}) > 0$ and 0 otherwise	Risk of hyperglycemia.
M value	$M = \frac{\sum_{t=t_i}^{t_k} \left\lvert 10 \times \log \frac{G_t \times 18}{\mathrm{IGV}} \right\rvert^3}{\mathrm{N}}$	G= glucose measured<br>IGV= ideal glucose value<br>$k =$ total numberof observations<br>$t_i =$ time in minutes after start of observations of the ith observation<br>$N =$ total number of readings	Indicator of glycemic control, and the stability of glucose excursions.
CONGA	$\sqrt{\frac{\sum_{t=t_i}^{t_k^*} (D - \overline{D})^2}{k^* - 1}}$<br>where<br>$D_t = GR_t - GR_{t-m}$ and $\overline{D} = \frac{\sum_{t=t_i}^{t_k^*} D_t}{k^*}$	$k^* =$ number of observations where there is an observation $n \times 60$ min ago<br>$m = n \times 60$<br>$D_t =$ difference between glucose reading at time t and t minus n hours ago	An objective assessment of glycemic variability over short time intervals.

SD, standard deviation; *CV,* coefficient of variation; *MAGE,* mean amplitude of glycemic excursions; *LBGI,* low blood glucose index; *HBGI,* high blood glucose index; *CONGA,* continuous overlapping net glycemic action; MODD, mean of daily difference; MAG, mean absolute glucose; ADRR, average daily risk range.

Reference：

1. Lin YH, Huang YY, Chen HY, Hsieh SH, Sun JH, Chen ST, et al. Impact of Carbohydrate on Glucose Variability in Patients with Type 1 Diabetes Assessed Through Professional Continuous Glucose Monitoring: A Retrospective Study. *Diabetes Ther*.（2019）10:2289-2304. doi:*10.1007/s13300-019-00707-x*
2. Joshi A, Mitra A, Anjum N, Shrivastava N, Khadanga S, Pakhare A, et al. Patterns of Glycemic Variability During a Diabetes Self-Management Educational Program. *Med Sci (Basel)*.（2019）7. doi:*10.3390/medsci7030052*
