# Supplementary material for: Effects of Variability in Glycemic Indices on Longevity in Chinese Centenarians
Source: Front Nutr. 2022 Jul 8;9:955101. doi: 10.3389/fnut.2022.955101 (PMC9307500; doi:10.3389/fnut.2022.955101)
Supplement: Supplementary file 2 [file Table_2.pdf]

Supplementary Table 2. **Characteristics of TBR in the different subgroups**

**Subgroup**	**Centenarian group (n=53)**	**First-generation offspring group (n=53)**	**Control group (n=20)**	***P***
3≤TBR<3.9	6.94% [1.46%,16.02%]	0.79% [0.00%-2.32%][a1]	1.47% [0.42%,5.23%][b2]	<0.001
TBR<3.0	0.21% [0.00%,1.22%]	0.00% [0.00%,0.05%][a1]	0.00% [0.00%,0.07%][b2]	<0.001

Annotations: a1, centenarian group *vs*. first-generation offspring group, $p<0.001$; a2, centenarian group *vs*. first-generation offspring group, $p<0.05$; b1, centenarian group *vs*. control group, $p<0.001$; b2, centenarian group *vs*. control group, $p<0.05$.
