# Supplementary material for: Effects of Variability in Glycemic Indices on Longevity in Chinese Centenarians
Source: Front Nutr. 2022 Jul 8;9:955101. doi: 10.3389/fnut.2022.955101 (PMC9307500; doi:10.3389/fnut.2022.955101)
Supplement: Supplementary file 3 [file Table_3.pdf]

Supplementary Table 3 TyG according to group assignment

Parameter	Centenarian group (n=53)	First-generation offspring group (n=53)	Control group (n=20)	***P***
TyG	2.63(1.91,3.45)	2.76(1.95,4.53)	2.73(1.70,4.74)	0.807

Supplementary Table 4 Logistic regression analysis of not achieving longevity and TyG

**Parameter**	**OR (95 % CI)1**	**P**	**OR (95 % CI)[2]**	**P**
TyG	1.37(1.01-1.85)	0.041	1.73(1.14,2.64)	0.010
